# Angiomyolipomes épithélioïdes rénal bénin: à propos de deux cas

**DOI:** 10.11604/pamj.2015.22.223.7975

**Published:** 2015-11-10

**Authors:** Tidiani Bagayogo, Yddoussalah Othmane, Karmouni Tarik, Elkhader Khalid, Koutani Abdellatif, Ibn Attya Andaloussi Ahmed

**Affiliations:** 1 CHU Ibn Sina, Service d'Urologie B, Rabat, Maroc

**Keywords:** Angiomyolipomes épithélioïdes, tumeur, rein, epithelioid angiomyolipoma, tumor, kidney

## Abstract

Les angiomyolipomes épithélioïdes rénaux (AMLeR) sont des tumeurs rares (identifiées chez moins de 0,1 patients pour 1000 habitants) et représentent 8% des angiomyolipomes (AML) opérés. Il a longtemps été considérécomme un hamartome mais plusieurs articles récents font penser qu'il s'agir d'une tumeur dérivant de cellules épithélioïdespérivasculaires. L'angiomyolipome épithélioïde est une forme rare d'angiomyolipome à potentiel malin, composé decellules épithélioïdes posant des problèmes de diagnostic différentiel avec les carcinomes à cellules rénales. L'immunohistochimie,en révélant la positivité des cellules épithélioïdes au marqueur HMB45 est essentielle au diagnostic. Les auteursrapportent l'aspect tomodensitométrique et histologique d'angiomyolipomes épithélioïdes chez deux patientes.

## Introduction

L'angiomyolipome est la tumeur mésenchymateuse la plus fréquente du rein [1]. Elle survient le plus souvent de façon sporadique, plus rarement dans le cadre d'une sclérose tubéreuse de Bourneville [2]. La variante épithélioïde de l'angiomyolipome (AML) a été décrite initialement par deux équipes en 1997 et 1998 [3, 4]. L'angiomyolipome épithélioïde rénal (AMLeR) est une tumeur rénale solide rare. L'AMLeR est une masse rénale solide qui atteint principalement les femmes (jusqu’à 78%) [5, 6]. La moyenne d’âge au diagnostic des AMLeR varie entre 38 et 50 ans [5, 7]. Le diagnostic d'AMLeR est obtenu sur pièce de néphrectomie et sur biopsie à l'aiguille fine. Nous rapportons dans cet article deux cas clinique d’ angiomyolipome épithélioïde rénal.

## Patient et observation

### Cas N° 1

**Figure 1:**
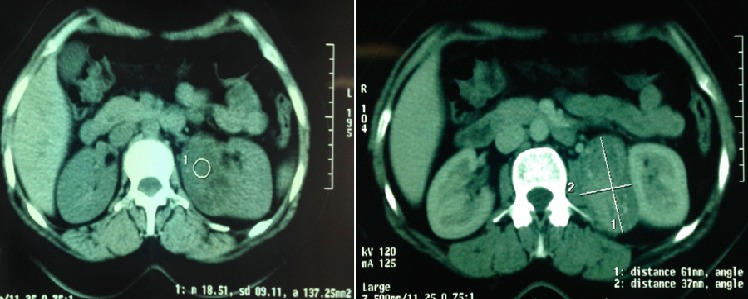
Coupe tomodensitométrique axiale mettant en évidence: (a) cliché au niveau du rein gauche sans injection avec une masse de lèvre inferieur du rein gauche, bien limite, heterodense. Ce processus mesure 61x37x51 mm de grand axe arrive au contact du psoas lombaire homolatéral et au contact intime avec l apophyse transverse de L2; (b) masse rénal se rehaussent après injection de produit de contraste

**Figure 2:**
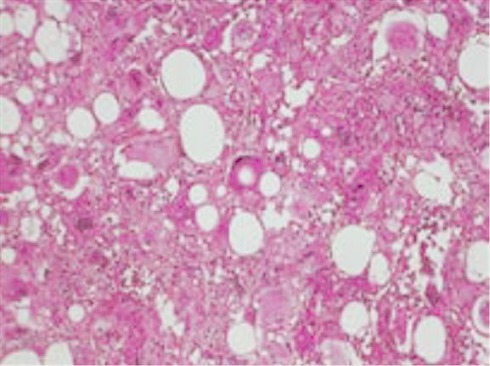
Angiomyolipome épithélioïde, cellules fusiformes et d'aspect épithélial de grande taille, HES × 40

Mme A.R, âgée de 35 ans,sans antécédent particulières.il se plaignait depuis 2 mois de lombalgies gauches de moyenne intensités associe a une seul épisode d hématurie, sans autre trouble urinaire, ou digestif associé. A l'examen clinique, la patiente a été apyrétique. Ses conjonctives ont été normalement colorées, et son abdomen souple. Les aires ganglionnaires ont été libres. La réalisation d'une échographie retrouvait une masse hyperéchogènes du rein gauche, conduisant à la réalisation d'un examen tomodensitométrique. La tomodensitométrie a objectivé une masse de lèvre inférieur du rein gauche, bien limite, heterodense, se rehaussent après injection de produit de contraste. Ce processus mesure 60 mm de grand axe arrive au contact du psoas lombaire homolatéral et au contact intime avec l apophyse transverse du corps vertébral de L2 (Figure 1). Sur le plan biologique, le patient a eu un taux d'hémoglobine à 11,3 g/dl, une fonction rénale normale avec une créatininémie à 7,5 mg.Le patient a été opérée par voie sous-costale gauche.Après décollement de l'angle colique gauche, il a bénéficié d'une néphrectomie partielle gauche. Les suites opératoires ont été simples. À l'examen macroscopique, masse tumoral solide mesurant 7 x 6 cm. A la coupe d aspect blanchâtre homogène. L'examen microscopique a mis en évidence une prolifération tumoral fait de trois composante: une composante vasculaire faite de vaisseaux a paroi épaissie autour des quels on note la présenced une deuxième composante fait de cellule épithélioïdes. Cette deuxième composante représente plus de 70% de la tumeur.la troisième composante est adipocytaire faite d adipocytes matures (Figure 2). Absence de mitoses atypiques. Cette analyse morphologique et histologique a conclu à un angiomyolipome épithélioïdes bénin du rein gauche.

### Cas N° 2

**Figure 3:**
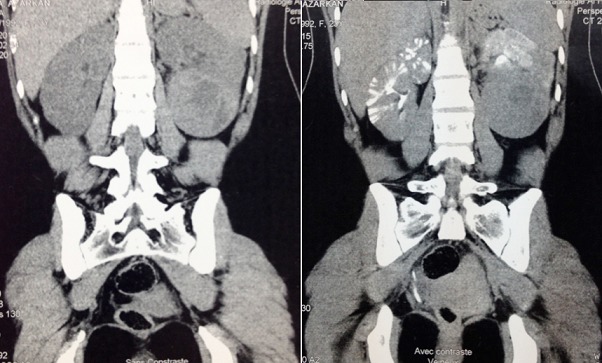
Coupe tomodensitométrique sagittale mettant en évidence: une masse tumorale du pole moyen et inferieur de 10 cm de grand axe, au contact intime avec le muscle psoas

**Figure 4:**
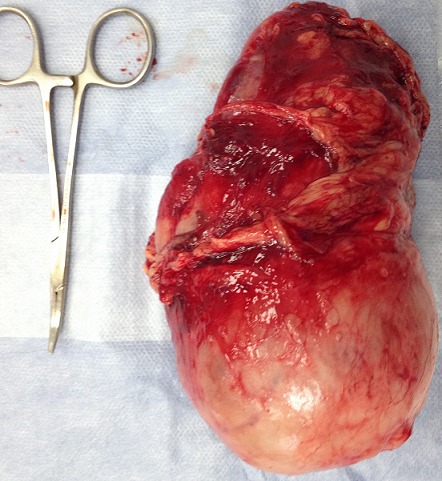
Aspect macroscopique de la pièce de néphrectomie: avec une masse tumoral polaire inferieur de 5x5x6 cm, situé a 1cm du hile

Mme A.A 24 ans, ayant consulté pour des lombalgies gauches associe à plusieurs épisodes d hématurie depuis six mois. A l'examen clinique il existait une masse Lombaire gauche difficilement palpable. L'uro-scanner objectivait un processus tissulaire partiellement nécrosé du pole inférieur mesurant dix centimètre de grand axe (Figure 3), pas d'envahissement de la veine rénale gauche ni de la veine cave inférieur. On note la présence de ganglions retropéritonéaux infra centimétrique. La patiente a été opérée par voie sous-costale elle a bénéficier d une néphrectomie gauche.Les suites opératoires ont été simples. L'examen macroscopique de la pièce opératoire montrait la coupe une tumeur polaire inférieur de 5x6 cm. la capsule rénale est bombée est en regard de la tumeur mais sans effraction capsulaire (Figure 4). En microscopie optique, il s agit d une prolifération tumorale faite de cellule polygonales de grande taille a cytoplasme abondant et oesinophile. les noyaux sont atypiques et irrégulières en forme et en taille. Cette tumeur infiltre la capsule par endroit sans la dépasser.la limite urétérale et le hile est indemne d infiltration tumorale. Cette analyse morphologique et histologique était en faveur d un angiomyolipome épithélioïdes bénin du rein gauche.

## Discussion

L'angiomyolipome est une lésion rare bien connue touchant les reins, le foie et d'autres organes d'AMLeR est morphologiquement très éloigné de l'angiomyolipome, classique.Le diagnostic histologique de cette variante et son rattachement à l'angiomyolipome, dont elle est morphologiquement très éloignée, n'ont pu être faits que par les études immunohistochimiques (négativité des cytokératines et positivité de l'HMB45). L'AMLeR c'est un PECome (PEC pour« perivascular epithelioid cell tumor ») de siège rénal ou pararénal de morphologie épithélioïde. Sur le plan clinique comme l'AML classique, l'AMLeR s'observe avec une égale fréquence chez l'homme et chez la femme, de préférence vers la quarantaine.Les AMLeR peuvent être sporadiques ou associés à la STB (de 31 à 50%) [5, 6]. Cette association est plus fréquente que l'association des AML classiques avec la STB (31% vs 20%) [5]. Les AMLeR sporadiques sont plus souvent symptomatiques que les cas associés à la STB [5]. Les masses non compliquées symptomatiques se manifestent par des douleurs du flanc chez 25% des patients ayant un AMLeR [5].

La tomodensitométrie (TDM) est le mode de révélation le plus fréquent des AMLeR [5]. Les AMLeR diagnostiqués histologiquement sont le plus souvent des AML pauvres en graisse à la TDM. L'AMLeR classique apparaît comme une masse arrondie, homogène isodense à la graisse, bien limité et à paroi fine. Aucun critère de densité ne permet d’établir le diagnostic Sûr d'AMLeR classique [5]. Le TDM thoraco-abdomino-pelvien est l'examen de référence Du bilan d'extension d'un AMLeR [8, 9]. Le bilan d'extension d'un AMLeR est indiqué en cas de suspicion de malignité ou dans le cadre d'une STB. Le TDM thoraco-abdomino-pelvien Permet de rechercher des localisations multiples ou à distance en fonction du potentiel de malignité. Une forme multiple d'AML doit faire rechercher une STB. Sur le plan macroscopique la tumeur est le plus souvent de grande taille (> 6 cm), compacte blanc grisâtre, mal limitée avec des remaniements hémorragiques. On évoque plus un carcinome rénal qu'un AML en l'absence de tissu adipeux reconnaissable. La tumeur peut être largement extériorisée par rapport au rein et ne lui être reliée que par un fin pédicule.

Microscopiquement,là aussi on évoque plutôt à première vue un carcinome rénal. La prolifération tumorale est faite de cellules fusiformes, ayant volontiers un cytoplasme abondant et clarifié, et de cellules d'allure épithéliale, globuleuses polygonales ou ovoïdes, éosinophiles de grande taille. On observe parfois des cellules géantes. Le plus souvent aucun secteur d'AML classique n'est observé [10, 11]. Les critères d'agressivité histologiquede l'AMLeR sont: une anaplasie nucléaire, une activité mitotique élevée (> 1/50 High Power Field (HPF), une invasionvasculaire, la présence de nécrose et une infiltration de lagraisse périrénale [7, 12]. Les marqueurs de différenciation mélanocytaires comme HMB-45 ou Melan-A en immunohistochimie sont positifs danstous les AML [5, 6]. Les trois composants de l'AML triphasique sont représentés dans des proportions variables: cellules musculaires Lisses, adipocytes et vaisseaux dystrophiques. Parmi les formes malignes de PEComes rapportées, les AMLeR malins rénaux sont les plus fréquents [12]. Avec quatre paramètres (clinique, TDM, anatomo-pathologique et bilan d'extension), la classification pronostique des PEComes [7] en AMLeR bénins, AMLeR à potentiel agressif et AMLeR malin permet la prise en charge. L'AMLeR bénin est défini par l'absence d'AEG ou L'absence d’évolution rapide, un aspect d'AML classique à la TDM rénale, une histologie non agressive et l'absence de localisation à distance.

La néphrectomie partielle devrait être indiquée pour les AMLeR bénins de plus de 4 cm en prévention des complications [5]. La néphrectomie partielle est le traitement De référence des AMLeR bénins quand il est techniquement réalisable, il permet le diagnostic anatomo-pathologique définitif, la prévention des complications, des récidives locales et les évolutions à distance [5]. Cependant, la néphrectomie totale peut être indiquée pour les AMLeR bénins de plus de 4 cm non ré-sécables en chirurgie partielle [5]. L'embolisation est le traitement des complications et de la prévention des complications pour les AMLeR bénins, à proposer dès 4 cm de façon préventive chez les patients non opérables. La surveillance devrait être indiquée pour les AMLeR localisés bénins de moins de 4 cm [5].

## Conclusion

L'AMLeR épithélioïde est une entité qui permet de rattacher à la famille des PEComes un certain nombre de tumeurs jusque là rattachées à des carcinomes ou même à des sarcomes. Pour les AMLeR bénins et les AMLeR multiples, le traitement est celui d'un AML typique.
